# Treatment continuation and factors associated with discontinuation with 3-month paliperidone palmitate in patients with schizophrenia: a *post hoc* analysis of post-marketing surveillance data in Japan

**DOI:** 10.3389/fpsyt.2026.1751249

**Published:** 2026-03-18

**Authors:** Yoshiteru Takekita, Natsuko Tokushige, Yosuke Saga

**Affiliations:** 1 Department of Neuropsychiatry, Faculty of Medicine, Kansai Medical University, Osaka, Japan; 2 Medical Affairs Division, Johnson & Johnson, Tokyo, Japan

**Keywords:** Japan, paliperidone palmitate 3-month formulation, *post hoc* analysis, schizophrenia, treatment continuation

## Abstract

**Introduction:**

Schizophrenia requires sustained treatment to prevent relapse and maintain functional stability. Poor adherence to oral antipsychotics is common and increases the risk of relapse and rehospitalization. Long-acting injectable antipsychotics, such as paliperidone palmitate 3-month formulation (PP3M), have been developed to improve adherence and clinical outcomes; however, evidence on clinical factors influencing treatment continuation and concomitant medication use in real-world settings remains limited. This *post hoc* analysis explored treatment continuation and its associated factors in Japanese patients with schizophrenia receiving PP3M, including reasons for discontinuation, mortality, and changes in concomitant medications.

**Methods:**

Data from a multicenter, prospective post-marketing surveillance in Japan were used. Patients stabilized on paliperidone palmitate 1-month formulation for ≥4 months were transitioned to PP3M. Treatment continuation was assessed using Kaplan–Meier analysis, and factors associated with discontinuation were evaluated using multivariable Cox regression analysis. Mortality rates and treatment-related standardized mortality ratios (SMRs) were calculated, and concomitant medication patterns were analyzed descriptively.

**Results:**

A total of 891 patients were included. The previously reported 12-month treatment continuation rate was 84.7%. Patients without concomitant medications had significantly higher continuation rates than those with concomitant medications (85.3% versus 76.8%, *P* = 0.001). The lowest starting dose of PP3M (175 mg) was a significant risk factor for discontinuation (hazard ratio 1.90, 95% confidence interval [CI] 1.12, 3.21). The treatment-related mortality was 3.94 per 1,000 person-years (95% CI 1.07, 10.09). The treatment-related SMR for the total population was 0.95 (95% CI 0.02, 1.89). Concomitant medication and benzodiazepine equivalent dose remained stable (treatment start, 35.79 mg; treatment end, 35.94 mg).

**Conclusions:**

PP3M demonstrated high treatment continuation in Japanese patients with schizophrenia, particularly those without concomitant medications. The lowest PP3M starting dose of 175 mg was associated with an increased risk of treatment discontinuation, underscoring the importance of individualized dosing strategies. Stable concomitant medication use supports symptomatic stability and the feasibility of PP3M monotherapy. These findings highlight the potential of PP3M to reduce pharmacologic burden and support long-term management of schizophrenia.

**Clinical trial registration:**

https://center6.umin.ac.jp/cgi-open-bin/ctr/ctr_view.cgi?recptno=R000047973, identifier UMIN000042033

## 1 Introduction

Schizophrenia is a chronic and disabling psychiatric disorder that is reported to affect 0.59%–0.7% of the Japanese population (1, 2). It is characterized by both positive symptoms (e.g., hallucinations and delusions) and negative symptoms (e.g., decreased motivation and social withdrawal), as well as cognitive impairments (e.g., deficits in attention, memory, and executive function) (3). Schizophrenia typically has its first onset during adolescence and young adulthood (1). When early intervention is delayed or insufficient, cognitive dysfunction may progress, interfering with attention, work capacity, and key aspects of life such as education, social participation, and career planning. This can lead to persistent functional challenges and diminished quality of life (QoL) (4). Although the incidence of new cases declines after young adulthood, prevalence peaks at around 40 years of age, reflecting the enduring nature of the illness and the accumulation of chronic cases over time (1, 5). The disease trajectory highlights the importance of sustained, individualized treatment strategies for long-term recovery and social reintegration.

Evidence suggests that early initiation of long-acting injectable (LAI) antipsychotics may provide substantial benefits beyond improving adherence, including greater symptomatic improvement, higher rates of sustained remission, and better functional outcomes in patients with shorter illness duration (6, 7). These findings support consideration of LAIs not only for patients with poor adherence, but also as an early treatment option to optimize long-term prognosis.

Antipsychotic medication remains the cornerstone of schizophrenia treatment and requires long-term management to prevent relapses and maintain functional stability. However, poor adherence to oral antipsychotics remains a challenge, with continuation rates in patients with schizophrenia as low as 40% (8). Discontinuation of antipsychotic therapy increases the risk of relapse by up to six times that of patients on continuous treatment (9). Meta-analytic data indicate markedly higher relapse after discontinuation than with maintenance, with pooled proportions around 57% versus 22% across studies irrespective of duration, and about 64% versus 27% at 7–12 months (10). Another meta-analysis reported that patients who discontinued antipsychotic medication had approximately double the risk of relapses compared with those who continued treatment (11). Relapse episodes are associated with negative outcomes, such as rehospitalization, deterioration of QoL, cognitive decline, and poorer long-term and functional prognosis (12, 13). The current requirement of long-term maintenance for most schizophrenia treatments coupled with low adherence rates for oral antipsychotic medications highlights the need for treatments with better adherence rates to prevent symptom relapse and rehospitalization.

To ensure effective prevention of relapse and rehospitalization in patients with schizophrenia, LAI antipsychotics have been recommended as a viable alternative to daily oral medications. The 2022 Japanese Society of Neuropsychopharmacology guidelines recommend the use of LAI in patients with poor adherence who are at risk of relapse, or when patients request it (3). Paliperidone palmitate 3‐month formulation (PP3M) is a long-acting, injectable, atypical antipsychotic that was approved for the treatment of schizophrenia in Japan in November 2020. The manufacturer’s instructions for use in Japan, per the package insert, indicate PP3M for the treatment of individuals with schizophrenia who have been stabilized with paliperidone palmitate 1‐month (PP1M) monotherapy without concomitant antipsychotics for at least 4 months and for whom safety and tolerability have been confirmed (14). Before switching to PP3M, an equivalent dose of PP1M must have been used in the previous two or more administrations.

Treatment indications differ between Japan and other countries. For example, in the United States, PP3M can be initiated after patients have been adequately treated with PP1M for at least 4 months, with the last two PP1M doses administered at the same dose level. Concomitant use of other antipsychotics is permitted. Following the initial PP3M dose, treatment should continue every 3 months, and if needed, dose adjustments may be made at 3-month intervals within the range of 273 mg to 819 mg based on individual tolerability and efficacy (15). In the EU, PP3M can be initiated in adult patients who have been adequately treated with PP1M (paliperidone palmitate monthly injection), preferably for 4 months or longer, without requiring prior dose adjustment. The initial PP3M dose then replaces the next scheduled PP1M dose and is determined using a 3.5-fold conversion factor from the previous PP1M dose. Thereafter, PP3M is administered every 3 months (± 2 weeks). If needed, dose adjustments may be made at 3-month intervals within the range of 175 mg to 525 mg based on individual tolerability and efficacy. Concomitant use of other antipsychotics is also permitted (16).

Several international real-world studies have reported higher rates of treatment continuation, adherence, relapse prevention, and rehospitalization prevention with PP3M compared with PP1M (17–19). The interim and final analyses of the multicenter, prospective, observational post-marketing surveillance (PMS) of PP3M conducted in Japan demonstrated its safety and effectiveness in Japanese patients with schizophrenia (20, 21). However, clinical factors influencing treatment continuation, mortality, and concomitant medication patterns in real-world settings remain underexplored. This *post hoc* analysis aimed to investigate reasons for discontinuation and to identify real-world clinical factors associated with treatment continuation, mortality rates, and changes in concomitant medication patterns among Japanese patients with schizophrenia treated with PP3M. To provide context, treatment continuation rate data from the previously reported pre-specified analysis are also included (21).

## 2 Methods

### 2.1 PMS design and study population

Details of the PMS design have been published in the primary analysis manuscript and are summarized here (21). In brief, the prospective, multicenter PMS was conducted across 201 sites in Japan between November 2020 and the end of May 2024 to evaluate the safety of PP3M (XEPLION TRI^®^ Aqueous Suspension for IM Injection). Patients were included in the PMS if they were diagnosed with schizophrenia and had been stabilized on PP1M for at least 4 months without concomitant antipsychotics, and received PP3M in accordance with the package insert. The initial dose of PP3M was determined based on the PP1M dose: if the PP1M dose is 50, 75, 100, or 150 mg, the initial dose of PP3M should be 175, 263, 350, or 525 mg, respectively, per the package insert (14).

The PMS included a 12-month observation period, with an additional 6-month extension (21). An electronic data capture system and central registration method were used to register patients and collect data. Data were collected over the 12-month observation period following the first PP3M injection and included baseline demographic and clinical characteristics (e.g., age, sex, employment status, cohabitation status, and comorbidities). Variables related to PP3M treatment included the starting dose, injection site, and reasons for switching. Data on concomitant medication, including benzodiazepines, non-benzodiazepine hypnotics, mood stabilizers, antidepressants, and antiparkinsonian drugs were also collected.

This PMS was conducted in accordance with the Japanese Good Post-Marketing Study Practice (Ministry of Health, Labor and Welfare [MHLW], Ministerial Ordinance No. 171 of 2004). Under the Good Post-Marketing Study Practice regulations, institutional review board approval and written informed consent for the PMS were not required; however, each participating institution obtained approval from an institutional review board if required by its own institutional procedures. This surveillance study was explained to patients by their physician, and only those who provided oral consent were registered. All patients enrolled in the study were provided with an explanation of the study either verbally or in written form. Patients who did not provide consent were excluded from participation.

### 2.2 Outcome measures

Outcome measures included factors associated with treatment continuation, overall mortality per 1,000 person-years, treatment-related mortality per 1,000 person-years, treatment-related standardized mortality ratio (SMR), concomitant medication patterns, and benzodiazepine dose equivalents. Treatment continuation, defined as the absence of PP3M discontinuation for any reason during the observation period, was evaluated as a pre-specified analysis and is reported in the PMS primary manuscript (21). Concomitant medications initiated after the start of PP3M treatment were documented and included in the analysis. The use of such medications was not regarded as indicative of PP3M discontinuation unless a switch to another antipsychotic agent occurred.

Overall mortality was defined as all-cause deaths occurring during the observation period, regardless of their relationship to PP3M treatment, and was expressed as a rate per 1,000 person-years. Treatment-related mortality was defined as deaths potentially associated with PP3M treatment, based on clinical evaluation, and was also expressed per 1,000 person-years. The SMR was calculated to assess mortality potentially related to PP3M and was defined as the ratio of the observed number of deaths to the expected number of deaths, with a reference value of 1.00. Mortality rates at each age were calculated using the MHLW abridged life tables (22).

### 2.3 Statistical analysis

Treatment continuation rates were analyzed using the Kaplan–Meier method. Descriptive statistics summarized baseline characteristics and reasons for discontinuation. A chi-square test was performed to compare the proportion of patients who discontinued treatment among patients who were versus were not receiving concomitant medications. A two-sided *P*-value was calculated. Odds ratios and 95% confidence intervals (CIs), which were based on a two-sided test, were calculated.

A multivariable Cox proportional hazards model was used to identify factors related to treatment discontinuation. Hazard ratios (HRs) and 95% CIs were calculated; covariates included sex, age, initial dose of PP3M, reason for switching to PP3M, PP3M administration site, presence of complications, concomitant medications, employment status, and cohabitation status. The mortality rate per 1,000 person-years and 95% CI were calculated using the number of deaths, patient-years, and mean treatment duration; overall mortality and treatment-related mortality rates were calculated separately.

Benzodiazepine equivalent doses were calculated using mean values, standard deviations (SDs), and ranges. Changes in concomitant medication use (benzodiazepines, non-benzodiazepine hypnotics, mood stabilizers, antidepressants, or antiparkinsonian drugs) were analyzed descriptively by comparing the number and percentage of patients receiving each medication category at the start of PP3M treatment and at the end of the treatment period.

Concomitant medications, excluding antipsychotic agents, were categorized into five distinct groups based on drug codes of Japan Standard Commodity Classification: benzodiazepines (code 1124); non-benzodiazepine hypnotics (codes 119 and 1129); mood stabilizers (lithium carbonate, code 1179017; antiepileptic drugs, code 113); antidepressants (imipramine derivatives, code 1174; other psychotropic agents, code 1179, excluding lithium carbonate); and antiparkinsonian drugs (code 116). The statistical analyses were conducted using SAS version 9.4 (SAS Institute Inc., Cary, NC, USA).

### 2.4 Ethical considerations

This *post hoc* analysis used anonymized data from a special drug use-results survey conducted in accordance with Good Post-Marketing Study Practice and approved by the Pharmaceuticals and Medical Devices Agency. Approval by an ethics review committee was considered unnecessary because no new intervention was involved.

## 3 Results

### 3.1 PMS patient characteristics and treatment patterns

A total of 891 patients with schizophrenia were included in the safety analysis set for both the primary study and this *post hoc* analysis. The PMS patient background and treatment status have been reported previously (21). In brief, the median (range) age was 51.0 (17.0–88.0) years, 18.9% of patients were aged ≥65 years, and 58.5% of patients were male. The PP3M starting dose was 175 mg, 263 mg, 350 mg, and 525 mg for 12.8%, 23.0%, 32.0%, and 32.2% of patients, respectively (21). The most common reasons for switching to PP3M were reduced administration burden (52.0%), fewer hospital visits (25.0%), and expectations of further effectiveness (19.0%) (21). Of the 891 patients, 438 (49.2%) did not receive any concomitant medications, and 453 (50.8%) were administered at least one concomitant medication (21).

### 3.2 Treatment continuation

**Figure 1:**
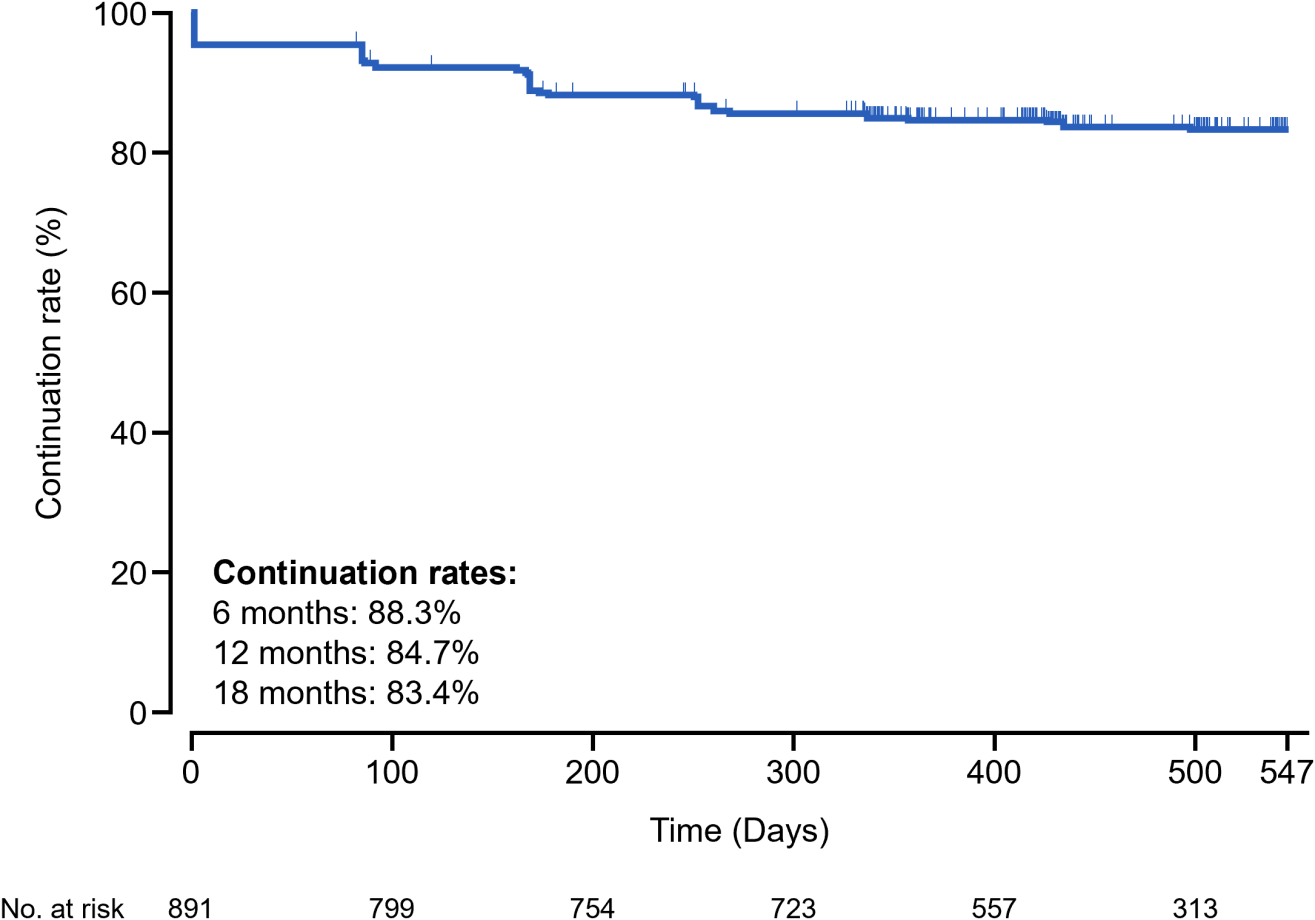
Kaplan–Meier curve of treatment continuation with PP3M over the observation period. The curve illustrates the cumulative probability of treatment continuation among 891 patients with schizophrenia enrolled in the Japanese post-marketing surveillance study. Vertical ticks indicate censored cases. Censoring occurred at the last recorded visit or upon discontinuation. The number at risk is shown at predefined time points. PP3M, paliperidone palmitate 3-month formulation.

**Figure 1** shows a Kaplan–Meier curve for treatment continuation with PP3M over the observation period. The 6-month treatment continuation rate was 88.3%. The 12- and 18-month treatment continuation rates previously reported were 84.7% and 83.4%, respectively (21).

Patients who did not receive any concomitant medications were more likely to continue PP3M treatment compared with those who did receive concomitant medications. Specifically, the treatment continuation rate was 85.3% (374/438) for patients without concomitant medications and 76.8% (348/453) for those with concomitant medications (two-sided *P* = 0.001). The odds of discontinuing treatment were 43% lower in patients without concomitant medications (odds ratio 0.57, 95% CI 0.40, 0.80). Of the 891 patients, 722 (81.0%) continued PP3M treatment throughout the observation period, while 169 (19.0%) discontinued. Among the 169 patients who discontinued PP3M, the main reasons were adverse events (35.5% [n = 60]), patient preference (18.9% [n = 32]), and hospital transfer (17.2% [n = 29]). Among the 60 patients who discontinued PP3M due to adverse events, the most commonly reported events were schizophrenia (21.7% [n = 13]), hyperprolactinemia (10.0% [n = 6]), insomnia (6.7% [n = 4]), and tremor (5.0% [n = 3]). The other adverse events were reported in only one or two patients.

### 3.3 Factors related to treatment continuation

Multivariable Cox proportional hazards analysis showed that the lowest dose of PP3M (175 mg) was significantly associated with a higher risk of treatment discontinuation (HR 1.90, 95% CI 1.12, 3.21; *P* = 0.017) (**Table 1**). No other factors, including sex, age, reason for switching to PP3M, administration site, presence or absence of complications, concomitant medications, employment status, or cohabitation status, were significantly associated with treatment discontinuation.

**Table 1:** Multivariable Cox regression analysis of treatment discontinuation predictors.

Characteristic		Multivariable analysis			
		n	HR	95% CI	*P*-value
Sex	Male	498	Reference	–	
	Female	359	1.25	0.86, 1.81	0.235
Age distribution	<30 years	60	Reference	–	
	30 to <40 years	122	0.90	0.41, 1.95	0.783
	40 to <50 years	216	0.74	0.36, 1.53	0.416
	50 to <60 years	233	0.62	0.30, 1.31	0.212
	≥60 years	226	0.54	0.25, 1.17	0.119
Initial dose of PP3M	525 mg	280	Reference	–	
	350 mg	270	1.10	0.70, 1.71	0.690
	263 mg	199	1.05	0.64, 1.74	0.839
	175 mg	108	1.90	1.12, 3.21	0.017
Reason for switching to PP3M	To reduce administration burden	450	Reference	–	
	To reduce hospital visits	219	0.86	0.55, 1.34	0.496
	To reduce financial burden	12	<0.01	–	0.978
	Expectation of further effectiveness	162	0.75	0.46, 1.22	0.240
	Expectation of reducing adverse events	14	1.40	0.43, 4.64	0.577
PP3M administration site	Deltoid muscle	334	Reference	–	
	Gluteal muscle	523	0.92	0.65, 1.31	0.650
Presence of complications	No	526	Reference	–	
	Yes	331	1.08	0.73, 1.58	0.708
Use of benzodiazepines	No	692	Reference	–	
	Yes	165	1.15	0.73, 1.80	0.551
Use of non-benzodiazepine hypnotics	No	657	Reference	–	
	Yes	200	0.98	0.63, 1.50	0.914
Use of mood stabilizers	No	735	Reference	–	
	Yes	122	1.04	0.63, 1.72	0.878
Use of antidepressants	No	800	Reference	–	
	Yes	57	1.61	0.89, 2.93	0.115
Use of antiparkinsonian drugs	No	782	Reference	–	
	Yes	75	1.24	0.70, 2.21	0.459
Employment status	Part-time/full-time worker	157	Reference	–	
	Student	14	2.70	0.86, 8.49	0.088
	In care	184	1.42	0.76, 2.63	0.272
	Unemployed	426	1.70	0.97, 2.99	0.065
	Full-time homemaker	76	1.32	0.57, 3.09	0.518
Cohabitation status	No	354	Reference	–	
	Yes	503	0.74	0.51, 1.09	0.132

CI, confidence interval; HR, hazard ratio; PP3M, paliperidone palmitate 3-month formulation.

### 3.4 Mortality analysis

The overall mortality rate was 14.78 per 1,000 person-years (95% CI 8.27, 24.38). The treatment-related mortality rate was 3.94 per 1,000 person-years (95% CI 1.07, 10.09), based on four deaths assessed as potentially associated with PP3M treatment. No deaths occurred among patients aged 10 to <50 years. The overall mortality rate included all deaths observed during the study period, regardless of their cause or relationship to PP3M treatment; in contrast, the treatment-related mortality rate only included deaths that were assessed as potentially associated with PP3M treatment, based on clinical evaluation. The treatment-related SMR for the total population was 0.95 (95% CI 0.02, 1.89). Age-stratified SMRs are presented in **Table 2**.

**Table 2:** Treatment-related SMRs at 1 year by age and sex.

Age at the start of treatment^a^		Male		Female		Total	
		SMR (95% CI)	SE	SMR (95% CI)	SE	SMR (95% CI)	SE
Overall		1.40 (0.03, 2.78)	0.70	0 (0.00, 0.00)	0.00	0.95 (0.02, 1.89)	0.48
Age category 1, years	50 to <60	2.08 (−2.00, 6.17)	2.08	0 (0.00, 0.00)	0.00	1.43 (−1.38, 4.24)	1.43
	60 to <70	1.28 (−1.23, 3.79)	1.28	0 (0.00, 0.00)	0.00	0. 90 (−0. 86, 2.66)	0.90
	70 to <80	2.15 (−0.83, 5.13)	1.52	0 (0.00, 0.00)	0.00	1.51 (−0.58, 3.60)	1.07
	≥80	0 (0.00, 0.00)	0.00	0 (0.00, 0.00)	0.00	0.00 (0.00, 0.00)	0.00
Age category 2, years	<65	0.95 (−0.91, 2.81)	0.95	0 (0.00, 0.00)	0.00	0.67 (−0.64, 1.97)	0.67
	≥65	1.67 (−0.22, 3.56)	0.96	0 (0.00, 0.00)	0.00	1.11 (−0.15, 2.37)	0.64

CI, confidence interval; SE, standard error; SMR, standardized mortality ratio.

^a^The age at the start of treatment was used to stratify survival up to 1 year from the start of treatment. Patients who died after Day 365 were censored at Day 365.

SMRs were calculated as the ratio of observed to expected deaths (reference = 1.00) based on these deaths.

### 3.5 Concomitant medication patterns

There were no substantial numerical changes in the number and proportion of patients receiving concomitant medications between the start and end of PP3M treatment (**Table 3**). Specifically, the use of benzodiazepines, non-benzodiazepine hypnotics, mood stabilizers, antidepressants, and antiparkinsonian drugs showed minimal variation throughout the treatment period.

**Table 3:** Concomitant medication patterns at baseline versus endpoint.

Concomitant medications^a^	Patients with concomitant medication, n (%) N = 891	
	At the start of PP3M administration	At the end of PP3M administration
Benzodiazepines	170 (19.1)	174 (19.5)
Non-benzodiazepine hypnotics	206 (23.1)	213 (23.9)
Mood stabilizers	126 (14.1)	122 (13.7)
Antidepressants	58 (6.5)	57 (6.4)
Antiparkinsonian drugs	78 (8.8)	75 (8.4)

PP3M, paliperidone palmitate 3-month formulation.

a Some patients used concomitant medications from more than one category.

The benzodiazepine equivalent dose (diazepam equivalent dose) remained stable during the study, with a mean ± SD benzodiazepine equivalent dose of 35.79 ± 95.53 mg at the start of PP3M treatment (median, 2.00 mg; range, 0.03–500.00 mg) and 35.94 ± 94.45 mg at the end of PP3M treatment (median, 2.00 mg; range, 0.03–500.00 mg).

## 4 Discussion

This *post hoc* analysis of the PMS study conducted in Japan demonstrated that patients without concomitant medications had significantly higher rates of treatment continuation than those with concomitant medications (85.3% versus 76.8%, *P* = 0.001), which translated to a 43% lower risk of discontinuation. Multivariable analysis identified the lowest starting dose of PP3M 175 mg as a significant risk factor for treatment discontinuation. The treatment-related mortality rate was 3.94 per 1,000 person-years, and the treatment-related SMR for the overall population was 0.95. Approximately half of the patients received concomitant medications, but no substantial changes were observed in their use throughout the treatment period. The mean benzodiazepine equivalent dose remained stable.

The findings of the PMS study provided real-world evidence that aligned with the Japanese label on treatment continuation with PP3M in Japanese patients with schizophrenia who transitioned from PP1M (21). The 12-month continuation rate reported for the PMS aligned with prior analyses (23).

Compared with PP1M, PP3M has an added advantage of increased adherence, likely because of the longer dose interval (12-month continuation rate: 69.1% versus 84.7%) (21, 24). The Japanese label requires patients to be treated with PP1M for at least 4 months before switching to PP3M (14). Although the duration of PP1M treatment before initiating PP3M treatment was not reported in the PMS, it was previously reported that PP3M continuation rates are significantly higher when the switch to PP3M follows >6 months of continuous treatment with PP1M (18), suggesting that stable symptom control by PP1M enables better treatment duration with PP3M. Better symptom control is expected to reduce relapses and rehospitalization (25–27). The higher treatment continuation rates among patients without versus with concomitant medications observed in the present *post hoc* analysis may be attributed to several factors, including patient demographics, how symptoms are controlled, and an increase in adverse effects from concomitant medications.

When assessed in the context of other LAI antipsychotics, these continuation rates are favorable. For olanzapine LAI, real-world data from the STAR study in Italy showed a 12−month continuation rate of 37.5%, with 62.5% of patients discontinuing treatment (28). Aripiprazole LAI showed a 12−month continuation rate of 76.5% in the schizophrenia subgroup of a UK real−world cohort (29) and 72.1% in a Korean cohort (30). For paliperidone palmitate 6−month formulation (PP6M), Najarian et al. (31) found that 87.0% of patients remained on treatment at 12 months, highlighting the strong long−term adherence associated with PP6M. These findings indicate that PP3M continuation rates observed in the present study (84.7% at 12 months and 83.4% at 18 months) are broadly consistent with or slightly higher than those reported for other LAIs, supporting its favorable profile for long–term management of schizophrenia.

The multivariable Cox regression analysis revealed that the lowest starting dose of PP3M (175 mg) was associated with an increased risk of treatment discontinuation (HR 1.90, 95% CI 1.12, 3.21; *P* = 0.017). Leucht et al. (32) previously reported that a risperidone-equivalent dose of 2.5 mg/day was sufficient to prevent relapse in patients in remission, but that risperidone-equivalent doses below 2.5 mg/day may increase relapse risks. Given that patients treated with PP3M have stable disease control, and 175 mg of PP3M is approximately equivalent to 2.0 mg/day of risperidone, our findings suggest that doses below this previously identified threshold may be linked to a higher risk of discontinuation due to inadequate effectiveness. No other clinical factors, including age, sex, or concomitant medication use, were significantly associated with discontinuation risk. These findings suggest that a dose sufficient to maintain relapse prevention may be necessary, highlighting the importance of tailoring dosing to each patient’s clinical condition to ensure adequate therapeutic effect and reduce the risk of early treatment discontinuation.

The proportion of patients receiving benzodiazepines did not notably change between the start of PP3M treatment and 12 months (19.1% and 19.5%, respectively), and was lower than that previously reported for patients taking PP1M (41.8%–43.2%) (24).

Although the mean ± SD benzodiazepine equivalent doses at the start of PP3M treatment (35.79 ± 95.53 mg) and at 12 months (35.94 ± 94.45 mg) were similar and notably higher than those reported with PP1M treatment (13.55 ± 12.35 mg and 13.03 ± 12.47 mg, respectively) (24), the median dose remained low at 2.00 mg and was wide ranging (0.03 to 500.00 mg). This indicates that a small number of patients received very high doses, which elevated the mean values, while most patients were prescribed relatively lower amounts. Therefore, the mean values may overrepresent actual usage because of the wide variability in dosing.

These findings suggest that the stability of concomitant medication use and benzodiazepine prescribing patterns may reflect overall symptomatic stability during PP3M treatment. Moreover, the absence of substantial changes in concomitant medication use indicates that switching to or continuing PP3M was not associated with new adverse events requiring additional pharmacological management.

Antipsychotic combination therapy is generally not recommended because it increases the risk of adverse events, complicates attribution of clinical benefit, and may worsen existing side effects. These practical and clinical concerns, together with the limited evidence supporting the efficacy of combination therapy, have resulted in most treatment guidelines recommending against its routine use. For example, the Japanese Society of Neuropsychopharmacology guideline emphasizes monotherapy as the standard approach and advises that combination therapy should only be considered in exceptional cases (3). Similarly, international guidelines, including those from the American Psychiatric Association (33), the National Institute for Health and Care Excellence (34), and the World Federation of Societies of Biological Psychiatry (35), support treatment with a single antipsychotic medication as the default approach. However, antipsychotic combination therapy may be considered predominantly in selected and exceptional clinical circumstances, such as treatment−resistant schizophrenia following an inadequate response to optimized clozapine treatment, or transiently during short−term cross−titration or the management of severe acute symptoms when clinically necessary, and should be time-limited, closely monitored, and regularly reassessed (34, 35).

Approximately half of the patients in this PMS were receiving PP3M without any concomitant medications, indicating that many patients were receiving PP3M monotherapy, which supports the value of PP3M as a viable treatment option. While the concomitant use of PP3M with other antipsychotics is permitted in some countries, such combinations are not recommended in Japan. This may reflect a unique treatment pattern for PP3M, particularly in the context of schizophrenia, where polypharmacy involving non-antipsychotic agents is common. Notably, the proportion of patients receiving concomitant benzodiazepines was lower with PP3M versus PP1M treatment (24), suggesting a reduction in the overall pharmacologic burden, which may be considered a benefit of PP3M. The findings from the present *post hoc* analysis clarify the current treatment landscape and demonstrate that PP3M monotherapy is achievable in real-world clinical practice.

Real-world evidence from other studies further contextualizes these findings. In a large US claims-based analysis of patients initiating PP3M, concomitant use of mental health-related medications during PP3M exposure was common: antidepressants (44.5%), oral atypical antipsychotics (37.4%), mood stabilizers (30.8%), and anxiolytics (21.2%) (36). Similarly, in the REMISSIO study, which evaluated PP3M in a real-world setting, 130 patients (42.6%) continued at least one psychotropic medication at baseline, and 92 patients (30.2%) initiated a new psychotropic medication during PP3M treatment; after the initiation of PP3M treatment until the end of the study, the most frequently used were lorazepam (5.2%), paliperidone (4.6%), risperidone (3.9%), olanzapine (3.6%), and biperiden (3.3%) (37). These findings indicate that, although PP3M monotherapy is feasible, concomitant psychotropic medication use remains prevalent in clinical practice, likely reflecting individualized treatment strategies for symptom control and comorbidities.

The PMS study had some limitations that also apply to this *post hoc* analysis (21). Although the transition to PP3M was confirmed to follow the method outlined in the prescribing information, the duration and continuity of PP1M administration prior to the switch were not verified. Given the nature of PMS studies, only patients for whom PP3M was deemed appropriate by the treating physician were included. Therefore, the findings may not be generalizable to the broader population of individuals with schizophrenia. Furthermore, as this *post hoc* analysis relied on data collected in routine clinical practice, missing data may have affected the robustness of the findings.

## 5 Conclusion

This *post hoc* analysis of a large-scale PMS study in Japan demonstrated high treatment continuation rates with PP3M among patients with schizophrenia who transitioned from stable PP1M treatment. The findings also revealed that nearly half of the patients were able to continue PP3M treatment without concomitant medications. The lowest starting dose of 175 mg was associated with a higher risk of treatment discontinuation, emphasizing the importance of individualized dosing strategies to maintain clinical stability and prevent relapse. These results highlight the potential of PP3M to reduce treatment burden, enhance treatment adherence, and support long-term disease management. Future prospective studies are warranted to validate these findings and further explore the impact of PP3M on patient-centered outcomes across diverse clinical populations.

## Data Availability

The datasets generated and analyzed for this study are not publicly available, as per confidentiality agreements with the participating medical institutions. Requests to access the datasets should be directed to the corresponding author.
